# Frequency Offset Tolerant Synchronization Signal Design in NB-IoT

**DOI:** 10.3390/s18114077

**Published:** 2018-11-21

**Authors:** Jun Zou, Chen Xu

**Affiliations:** Ministerial Key Laboratory of JGMT, Nanjing University of Science and Technology, Nanjing 210094, China; chenxu_njust@163.com

**Keywords:** Internet of Things, synchronization signal, timing detection, large frequency offset

## Abstract

Timing detection is the first step and very important in wireless communication systems. Timing detection performance is usually affected by the frequency offset. Therefore, it is a challenge to design the synchronization signal in massive narrowband Internet of Things (NB-IoT) scenarios where the frequency offset is usually large due to the low cost requirement. In this paper, we firstly proposed a new general synchronization signal structure with a couple of sequences which are conjugated to remove the potential timing error that arises from large frequency offset. Then, we analyze the suitable sequence for our proposed synchronization signal structure and discuss a Zadoff–Chu (ZC) sequence with root 1 as an example. Finally, the simulation results demonstrate that our proposed synchronization signal can work well when the frequency offset is large. It means that our proposed synchronization signal design is very suitable for the massive NB-IoT.

## 1. Introduction

Internet of Things (IoT) is a network that aims to connect all the devices in the world and makes the information exchange between them easily. With the rapid development of wireless communication technologies, the IoT has been realized step by step in recent years. Over 60% of the IoT application scenarios are massive narrowband IoT (NB-IoT), whose characteristics include massive access devices, transmission delay tolerance and low cost, such as smart meters, eHealth, and so on [1,2,3,4,5]. The low-cost requirement means that cheap/inaccurate crystal oscillators are used, which leads to a large frequency offset between the transmitter and the receiver. It poses challenges to the design of the downlink synchronization signal which is used to obtain the downlink timing. In a traditional Long Term Evolution (LTE) system, the primary synchronization signal (PSS) and the secondary synchronization signal (SSS) are used to achieve the downlink timing [6,7]. PSS detection, as the very first process, usually suffers from the large frequency offset. The frequency offset will destroy the ‘perfect’ correlation of the Zadoff–Chu (ZC) sequence used by the PSS, which could lead to a timing error even if there is no noise [7]. Then, the differential correlator or partial correlator is usually used to remove or diminish the effects of the frequency offset on timing detection [8].

In this paper, we firstly proposed a new general synchronization signal structure with two PSS sequences in an adjacent period which are conjugated to remove the potential timing error caused by the frequency offset. Then, we analyze the suitable sequence for our proposed structure and analyze a special ZC sequence as an example. Finally, simulation results are given to verify our proposed design.

## 2. System Model

Assume that the synchronization signal in time domain is x[n],  n=0,1,⋯,N−1 at the transmitter, where N is the length of the synchronization sequence. Assuming there is one receiving antenna, the received signal at the device can be written as follows:

(1) y[n]=hx[n−d]+w[n] 

where $h\sim CN(0,\;\sigma_{h}^{2})$ is the channel gain, unknown but assumed to be constant over the PSS transmission duration, d is the number of samples corresponding to the signal propagation time between the transmitter and the receiver, and $w[n]\sim CN(0,\sigma_{w}^{2})$ is the white Gaussian noise.

When considering the frequency offset, the received signal at the device can be rewritten as follows:

(2) $$y[n]=hx[n-d]e^{j2\pi n\Delta f/f_{s}}+w[n],$$

where $f_{s}$ is the Nyquist sampling rate without a consideration of the sampling offset and Δf is the frequency offset between the transmitter (base station) and the receiver (device).

At the receiver, a local sequence which is a copy of the transmitter synchronization sequence is used to detect the right receiving start time by calculating the correlation between the received signal and the local sequence. The output of the direct correlator can be written as follows [9]:

(3) $$z(k)=\frac{1}{N}\sum_{n=0}^{N-1}y[n+k]x^{*}[n]\;$$

When the phase change caused by the frequency offset is large in the signal duration, e.g., $\frac{2\pi \Delta fN}{f_{s}}>\pi$, the M-part correlator or differential correlator will be used to replace the direct correlator [8]. Just as the name implies, the purpose of the M-part correlator is to divide the sequence into M parts while doing correlation operations. M is decided by the maximum phase shift, and the relationship can be written as follows:

(4) $$2\pi N\Delta f/(f_{s}\cdot M)<\varphi \;$$

where φ is the threshold to limit the phase change in each part; its value depends on its specific applied environment. Then, we can work out the number of segments:

(5) $$M=\left\lceil \frac{2\pi N\Delta f}{\varphi f_{s}}\right\rceil \;$$

⌈·⌉ is the integer-valued function. Obviously, the maximum phase in each part will decrease as the number of segments increase, which means stronger frequency offset tolerance. The output of the M-part correlator can be written as follows:

(6) $$z(k)=\frac{1}{N}\sqrt{{\sum_{m=0}^{M-1}\left|\sum_{n=\frac{N}{M}m}^{\frac{N}{M}(m+1)-1}y[n+k]x^{*}[n]\right|}^{2}}\;$$

Obviously, the processing gain will decrease approximately $10\log_{10}M$ dB for the M-part correlator due to the non-coherent combination among the different parts compared to the direct correlator. 

The output of the differential correlator can be expressed as follows [10]:

(7) $$z(k)=\frac{1}{N-k_{d}}\sum_{n=0}^{N-1-k_{d}}y[n+k_{d}+k]y^{*}[n+k]x^{*}[n+k_{d}]x[n]\;$$

Although the differentiation operation can remove the frequency offset perfectly, there will be at least a 3 dB loss in the signal-to-noise ratio (SNR) due to the fact that the noise is amplified by the multiplication between two received samples [5]. Therefore, these two methods are usually used when the frequency offset is large.

Then, a maximum likelihood estimate (MLE) is used to estimate the right timing, and it can be expressed as follows [9]:

(8) $$\hat{k}=\underset{k}{\arg\max}\left|z(k)\right|\;$$

## 3. Conjugated-Sequences-Based Timing Structure

Our proposed synchronization signal consists of a couple of sequences which are conjugated. When we ignore the noise and the channel gain (constant in one detection process), the output of the direct correlator in (3) can be rewritten as follows:

(9) $$\begin{array}{cc} \left|z_{1}(\Delta k_{1},\Delta f)\right| & =\left|\frac{1}{N}\sum_{n=0}^{N-1}x[n+k_{1}-d]e^{j2\pi (n+k_{1})\Delta f/f_{s}}x^{*}[n]\right|\\ & =\frac{1}{N}\left|\sum_{n=0}^{N-1}x[n+\Delta k_{1}]x^{*}[n]e^{j2\pi n\Delta f/f_{s}}\right| \end{array}$$

where $\Delta k_{1}≜k_{1}-d$, $k_{1}$ is the index of the direct correlator output with local sequence x[n]. 

The other synchronization sequence in our design is $x^{*}[n],\;n=0,1,\cdots ,N-1$. The corresponding correlator output can be expressed as follows:

(10) $$\begin{array}{cc} \left|z_{2}(\Delta k_{2},\Delta f)\right| & =\left|\frac{1}{N}\sum_{n=0}^{N-1}x^{*}[n+k_{2}-d]x[n]e^{j2\pi (n+k_{2})\Delta f/f_{s}}\right|\\ & =\frac{1}{N}\left|\sum_{n=0}^{N-1}x[n]x^{*}[n-\Delta k_{2}]e^{j2\pi n\Delta f/f_{s}}\right| \end{array}$$

where $\Delta k_{2}≜d-k_{2}$, $k_{2}$ is the index of the direct correlator output with local sequence $x^{*}[n]$. 

Obviously, when $\Delta k_{1}=\Delta k_{2}$

(11) $$\left|z_{1}(\Delta k_{1},\Delta f)\right|=\left|z_{2}(\Delta k_{2},\Delta f)\right|\;$$

without considering the data adjacent to the synchronization sequence, that is

(12) x[n]=0, n≥N or n<0  

It means if the timing estimated from the correlator with local sequence x[n] is

(13) $${\hat{k}}_{1}=\Delta {\hat{k}}_{1}+d\;$$

the timing estimated from the correlator with local sequence $x^{*}[n]$ must happen at

(14) $${\hat{k}}_{2}=d-\Delta {\hat{k}}_{2}=d-\Delta {\hat{k}}_{1}\;$$

where $\Delta {\hat{k}}_{1}$ and $\Delta {\hat{k}}_{2}$ are the timing estimation errors which are the same according to (11).

Therefore, the right timing can be estimated according to

(15) $$\hat{k}=\frac{{\hat{k}}_{1}+{\hat{k}}_{2}}{2}\;$$

which can remove the potential reducible timing error caused by the large frequency offset which is difficult to avoid [11]. 

**Figure 1:**
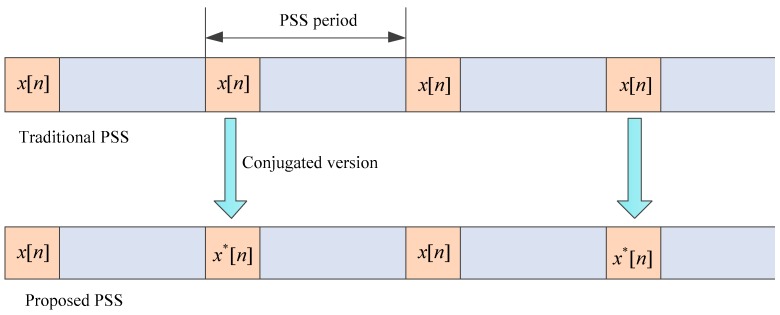
Illustration of our proposed conjugated-sequences-based primary synchronization signal (PSS) structure.

In the current LTE system, the synchronization signal is periodic, so we let the two PSS sequences in the adjacent period utilize the x[n] and $x^{*}[n]$, respectively, as shown in Figure 1. When the timing error caused by the frequency offset is small (for the device with an expensive crystal oscillator), it needs to search just one of the PSS signals rather than two for the purpose of a quick search, low complexity, and compatibility. In addition, it can maintain the peak-to-average power ratio (PAPR) property of the original sequence x[n] without the need of PAPR reduction techniques [12,13,14,15]. 

Obviously, the key of our proposed design is to find a sequence whose maximum correlator output is insensitive to the frequency offset.

## 4. Frequency Offset Tolerant Signal Selection

Accord to the Cauchy–Schwartz inequality [16], the output of the direct correlator from Equation (9) satisfies

(16) $$\left|\frac{1}{N}\sum_{n=0}^{N-1}x[n+\Delta k_{1}]x^{*}[n]e^{\frac{j2\pi n\Delta f}{f_{s}}}\right|\leq \frac{1}{N}\sqrt{\sum_{n=0}^{N-1}{\left|x[n+\Delta k_{1}]\right|}^{2}\sum_{n=0}^{N-1}{\left|x^{*}[n]\right|}^{2}}\;$$

where ${\sum_{n=0}^{N-1}\left|x[n+\Delta k_{1}]\right|}^{2}={\sum_{n=0}^{N-1}\left|x^{*}[n]\right|}^{2}$ is the energy of the synchronization signal. The Equation (16) holds if and only if

(17) $$x[n+\Delta k_{1}]=Cx[n]e^{\frac{j2\pi n\Delta f}{f_{s}}},$$

where C is constant.

For the consideration of good PAPR, the amplitude of x[n] should be constant (|C|=1). We assume

(18) $$x[n]=e^{j\pi \sum_{i=0}^{K-1}a_{i}n^{i}}.$$

Substituting (8) into (17) yields

(19) $$\sum_{i=0}^{k-1}a_{i}{\left(n+\Delta k_{1}\right)}^{i}=\sum_{i=0}^{k-1}a_{i}n^{i}+2n\Delta f/f_{s}\;$$

By comparing the coefficient of $n^{i}$ on both sides of the equation, we can obtain

(20) $$x[n]=e^{j\pi (a_{2}n^{2}+a_{1}n+a_{0})}.$$

Then, substituting (20) into (19) yields

(21) $$\begin{array}{l} \left|z_{1}(\Delta k_{1},\Delta \lambda ,a_{2})\right|\\ =\frac{1}{N}\left|\frac{\sin\left(\frac{\pi \Delta \lambda (N-\left|\Delta k_{1}\right|)}{N}+\pi a_{2}\Delta k_{1}(N-\left|\Delta k_{1}\right|)\right)}{\sin\left(\frac{\pi \Delta \lambda }{N}+\pi a_{2}\Delta k_{1}\right)}\right| \end{array}$$

where $\Delta \lambda =\Delta f/\Delta f_{s}$ is the frequency offset normalized to the subcarrier spacing $\Delta f_{s}$. According to the property of the sine function, (21) can be rewritten as

(22) $$\begin{array}{l} \left|z_{1}(\Delta k_{1},\Delta \lambda ,a_{2})\right|\\ =\frac{1}{N}\left|\frac{\sin\left(\frac{\pi \Delta \lambda (N-\left|\Delta k_{1}\right|)}{N}+\pi \xi \Delta k_{1}(N-\left|\Delta k_{1}\right|)\right)}{\sin\left(\frac{\pi \Delta \lambda }{N}+\pi \xi \Delta k_{1}\right)}\right| \end{array}$$

where $\xi =a_{2}-\left\lfloor a_{2}\right\rfloor$, ⌊⋅⌋ is the integer-valued function. It means the correlator output only depends on the fractional part of coefficient $a_{2}$ that corresponds to the sequence. For ease of discussion, we can only consider the value of $a_{2}\in$[0,1).

From the previous analysis, we know that the ZC sequence is one of the sequences that satisfies the constraints and is suitable for our proposed PSS design. It can be expressed as [17]

(23) $$x[n]=e^{-j\frac{\pi \mu n(n+1)}{N}},\;n=0,1,2,\cdots N-1\;$$

where N is the length of the ZC sequence, μ=0,1,2,⋯,N−1 is the root of the ZC sequence and $a_{2}=\frac{\mu }{N}$.

**Figure 2:**
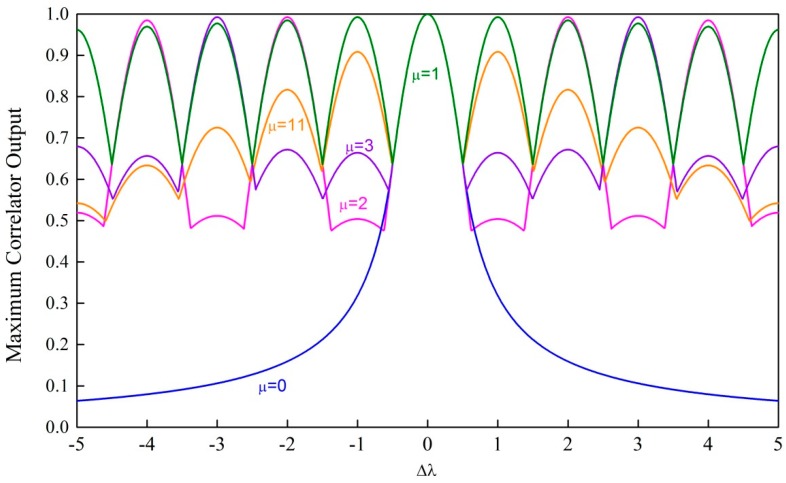
Illustration of the maximum correlator output of Zadoff–Chu (ZC) sequences with different roots (N=131).

Apparently, the correlator outputs of the ZC sequences with different roots are different. In addition, the low reduction of maximum output of the correlator under large frequency offsets is beneficial to our proposed method. Figure 2 shows an example of the maximum correlator output of different ZC sequences (e.g., N=131) under different frequency offsets, where different colors represent ZC sequences with different roots. The maximum output of the correlator is defined as

(24) $$z_{\max}(\Delta \lambda ,a_{2})=\underset{\Delta_{k_{1}}}{\max}\left|z_{1}(\Delta k_{1},\Delta \lambda ,a_{2})\right|\;$$

From Figure 2, we can see that the ZC sequence with root μ=1 is most insensitive to the frequency offset. When μ=1, (22) can be rewritten as

(25) $$\left|z_{1}(\Delta k_{1},\Delta \lambda ,\frac{1}{N})\right|=\{\begin{array}{cc} \frac{1}{N}\left(N-\left|\Delta \lambda \right|\right) & \Delta k_{1}=-\Delta \lambda \\ \frac{1}{N}\left|\frac{\sin\left(\frac{\pi (\Delta \lambda +\Delta k_{1})(N-\left|\Delta k_{1}\right|)}{N}\right)}{\sin\left(\frac{\pi (\Delta \lambda +\Delta k_{1})}{N}\right)}\right| & others \end{array}\;$$

**Figure 3:**
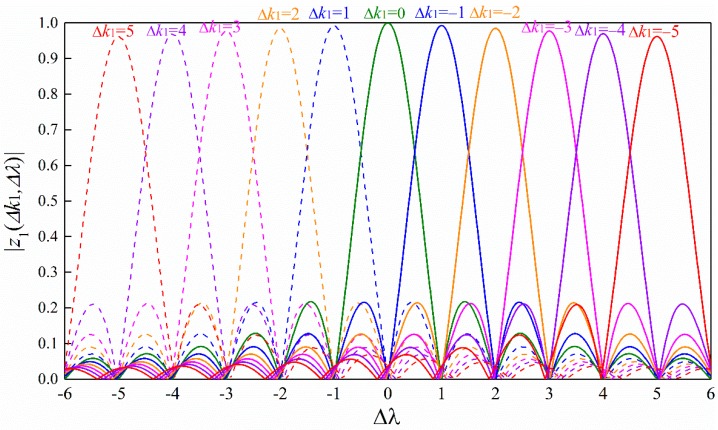
Illustration of the correlator output of the ZC sequence with root μ=1 under different timing offsets.

Figure 3 shows the correlator output in (25) under different frequency offsets and different timing offsets with μ=1. We can see that there is no timing error when the frequency offset is small (e.g., Δλ<0.5). However, when the frequency offset increases, the timing error is present according to the criterion defined in (8). However, the timing error can be removed easily by our proposed design. In addition, by comparing Figure 2 and Figure 3, we can see that the minimum of the maximum correlator output decreases slowly when the frequency offset Δλ increases step by 1. This is because the gap of the timing offset $\Delta k_{1}$ corresponding to the adjacent peaks is one which is always true with μ=1 for arbitrary N. Therefore, the ZC sequence with μ=1 is suitable for our proposed conjugated-sequences-based timing structure.

According to (25) and Figure 3, we can know that the minimum of the maximum correlator output happens at the cross point corresponding to the adjacent timing offset. When $\Delta k_{1}\geq 0$, $\Delta \lambda \in [-\Delta k_{1}-\frac{1}{2},-\Delta k_{1}+\frac{1}{2}]$, it is easy to show that

(26) $$\left|z_{1}(\Delta k_{1},\Delta \lambda ,\frac{1}{N})\right|>\left|z_{1}(\Delta k_{1}+1,\Delta \lambda -1,\frac{1}{N})\right|\;$$

according to (25). When $\Delta k_{1}\leq 0$, Δλ∈ $\left[\Delta k_{1}-\frac{1}{2},\Delta k_{1}+\frac{1}{2}\right]$, it is also easy to show that

(27) $$\left|z_{1}(\Delta k_{1},\Delta \lambda ,\frac{1}{N})\right|>\left|z_{1}(\Delta k_{1}-1,\Delta \lambda +1,\frac{1}{N})\right|\;$$

**Figure 4:**
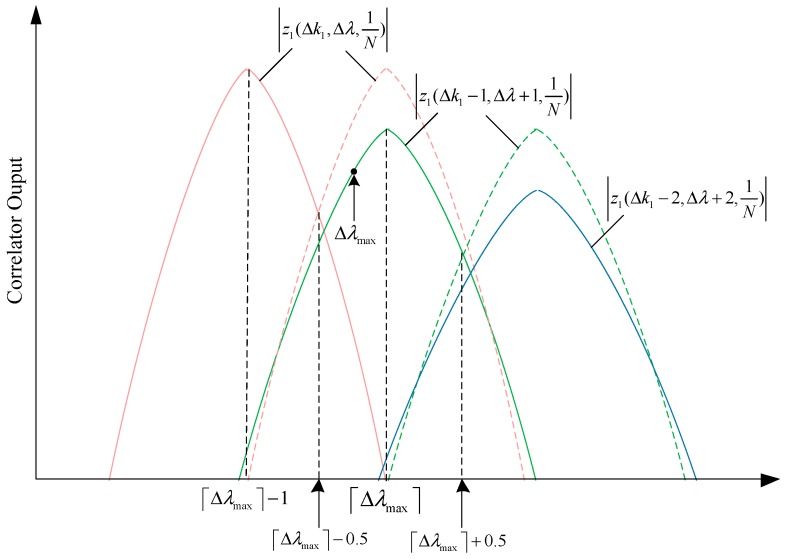
Illustration of the main lobes of the correlator output around the maximum frequency offset $\Delta \lambda_{\max}$ ($\Delta \lambda_{\max}$ > 0 in this example). The dash line is the copy of the solid one with the same color.

According to (26), (27) and Figure 4, we can get that at the given maximum frequency offset $\Delta \lambda_{\max}$, the minimum of the maximum correlator output satisfies

(28) $$z_{\min}>\left|z_{1}(-\left\lceil \left|\Delta \lambda_{\max}\right|\right\rceil ,\left\lceil \left|\Delta \lambda_{\max}\right|\right\rceil +0.5,\frac{1}{N})\right|=\frac{1}{N}\left|\frac{\sin\left(\frac{\pi (N-\left\lceil \left|\Delta \lambda_{\max}\right|\right\rceil )}{2N}\right)}{\sin\left(\frac{\pi }{2N}\right)}\right|\;$$

The current narrowband PSS (NPSS) in NB-IoT is about 0.8 ms in time domain [18], so the subcarrier spacing in our proposed PSS is 1/0.8 ms=1.25 kHz and N=131 for the justice comparison. When the maximum frequency offset is 40 kHz, which is equal to 20 ppm at the carrier frequency 2 GHz, the maximum detection energy loss is approximately

(29) $$10\log_{10}\frac{\sin\left(\frac{\pi (N-\left\lceil \left|\Delta \lambda_{\max}\right|\right\rceil )}{2N}\right)}{N\sin\left(\frac{\pi }{2N}\right)}\approx -2.3\;dB\;$$

which is smaller than the energy loss in the differential correlator and M-part correlator.

Therefore, the ZC sequence with root μ=1 is very suitable for our proposed conjugated-sequences-based PSS structure whose correlator output is insensitive to frequency offset.

## 5. Simulation Results

In this section, simulation results are given to verify the performance of our proposed PSS signal with a couple of ZC sequences which are conjugated. The system parameters are set as follows: the proposed PSS is generated in a time domain in the orthogonal frequency division multiplexing (OFDM) system, the subcarrier spacing is $\Delta f_{s}=1.25$ kHz, the length of ZC sequence is N=131, the root of ZC sequence is μ=1, and the synchronization signal period is 10 ms (the same as the NPSS in NB-IoT [18]). In the simulation, the frequency offset is randomly and uniformly selected and added. The additive white Gaussian noise (AWGN) channel is used in the simulations. When the timing error is larger than 1 us, we regard it as detection error. 

**Figure 5:**
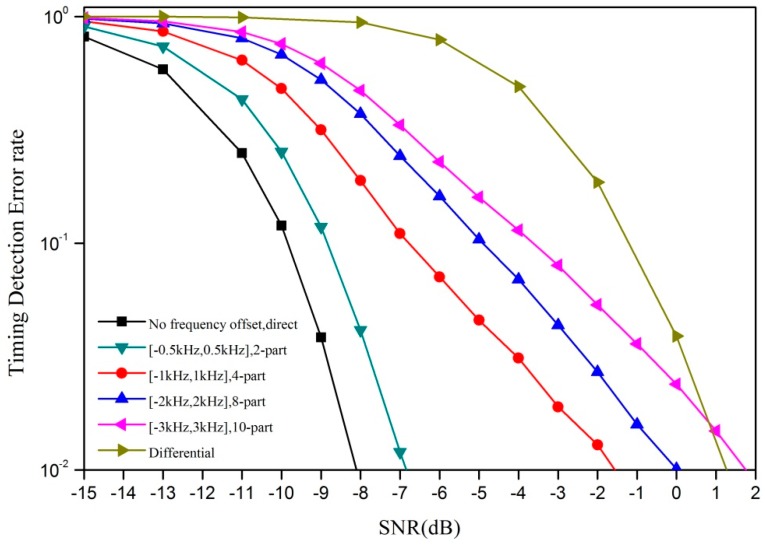
Timing detection error rate of the M-part correlator and the differential correlator with different frequency offsets.

Figure 5 shows a contrast of the timing detection error rate between the M-part correlator and the differential correlator. We can see that 3 kHz is a cut-off point between the M-part correlator and the differential correlator. In low frequency offset scenarios, thanks to the reduction of phase change in each segment, the M-part correlator works better than the differential correlator. However, when the frequency offset is larger than 3 kHz or more, the M-part correlator has to enlarge M in order to ensure that each part’s phase change is small enough. A larger M means a lower correlation peak, so its performance largely depends on noise. In this scenario, differential correlator is evidently a better choice.

**Figure 6:**
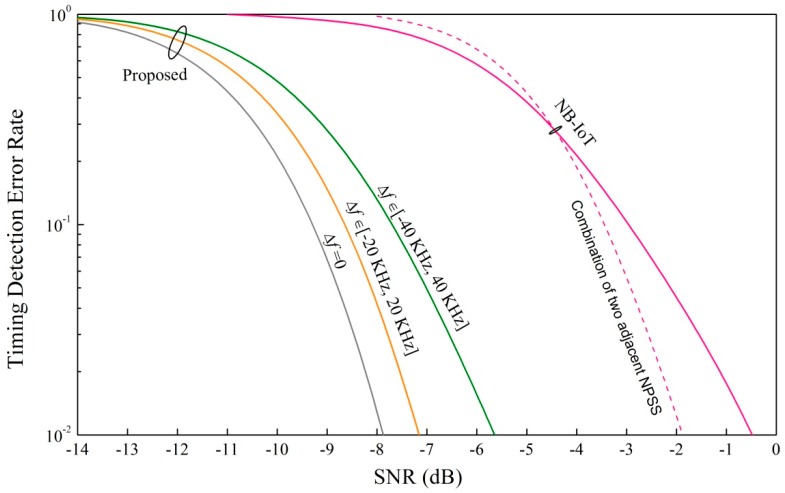
Timing detection error rate of our proposed synchronization signal with different frequency offsets.

Figure 6 shows the timing detection error rate of our proposed PSS at different SNRs. We also add the NB-IoT NPSS detection performance as a reference where the differential correlator is used [19]. For a fair comparison, we also simulated the timing performance of the combination of two adjacent NPSS that the estimated timing is the average of the timing estimated from the two adjacent NPSS. We can see that the deterioration of the timing performance caused by the frequency offset is small because the correlator output of the ZC sequence with root 1 is insensitive to the frequency offset. We can see that our proposed synchronization signal can work better than the NPSS with a differential correlator even under the maximum frequency offset 40 kHz. It is true that the detection performance of our proposed synchronization signal will be worse than the NB-IoT PSS with the increment of frequency offset due to the fact that the differentiation operation can remove the frequency offset effect on detection. However, our proposed PSS design is good enough to cover the potential frequency offset range in NB-IoT scenarios (less than 40 kHz [20]).

## 6. Conclusions

This paper investigates the downlink synchronization signal design in a large frequency offset scenario. We propose a general synchronization signal structure design with a couple of sequences which are conjugated to deal with the timing issue instead of the differentiation operation or partial correlator. Moreover, we discuss the general formulae of the suitable sequence for our proposed structure. ZC sequence with root 1 is analyzed as an example to verify the feasibility of the proposed conjugated structure. The loss of the detection energy is small due to the special ZC sequence’s insensitivity to frequency offset. The simulation results demonstrate that our proposed synchronization signal can work better than the NPSS under large frequency offset. From the simulation results, timing detection performance loss is approximately 2 dB when the frequency offset is in the range of −40 kHz through 40 kHz. Therefore, our proposed synchronization signal is very suit for the low cost NB-IoT scenarios.
